# Protocol for the atWork trial: a randomised controlled trial of a workplace intervention targeting subjective health complaints

**DOI:** 10.1186/s12889-016-3515-x

**Published:** 2016-08-19

**Authors:** Tone Langjordet Johnsen, Aage Indahl, Valborg Baste, Hege Randi Eriksen, Torill Helene Tveito

**Affiliations:** 1 Division of Physical Medicine and Rehabilitation, Vestfold Hospital Trust, POB 2168, NO 3103 Tønsberg, Norway; 2 Uni Research Health, POB 7810, NO 5020 Bergen, Norway; 3 Department of Sport and Physical Activity, Bergen University College, Bergen, Norway; 4 Department of Health Promotion, University College of Southeast Norway, Horten, Norway

**Keywords:** Sick leave, Subjective health complaints, Mental health complaints, Musculoskeletal complaints, Back pain, Anxiety, Depression, Coping, Workplace intervention, Randomised controlled trial

## Abstract

**Background:**

Subjective health complaints, such as musculoskeletal and mental health complaints, have a high prevalence in the general population, and account for a large proportion of sick leave in Norway. It may be difficult to prevent the occurrence of subjective health complaints, but it may be possible to influence employees’ perception and management of these complaints, which in turn may have impact on sick leave and return to work after sick leave. Long term sick leave has many negative health and social consequences, and it is important to gain knowledge about effective interventions to prevent and reduce long term sick leave.

**Methods/Design:**

This study is a cluster randomised controlled trial to evaluate the effect of the modified atWork intervention, targeting non-specific musculoskeletal complaints and mental health complaints. This intervention will be compared to the original atWork intervention targeting only non-specific musculoskeletal complaints. Kindergartens in Norway are invited to participate in the study and will be randomly assigned to one of the two interventions. Estimated sample size is 100 kindergartens, with a total of approximately 1100 employees. Primary outcome is sick leave at unit level, measured using register data from the Norwegian Labour and Welfare Administration. One kindergarten equals one unit, regardless of number of employees. Secondary outcomes will be measured at the individual level and include coping, health, job satisfaction, social support, and workplace inclusion, collected through questionnaires distributed at baseline and at 12 months follow up. All employees in the included kindergartens are eligible for participating in the survey.

**Discussion:**

The effect evaluation of the modified atWork intervention is a large and comprehensive project, providing evidence-based information on prevention of long-term sick leave, which may be of considerable benefit both from a societal, organisational, and individual perspective.

**Trial registration:**

Clinicaltrials.gov: NCT02396797. Registered March 23th, 2015.

## Background

Subjective health complaints (SHC), such as musculoskeletal and mental health complaints, have a high prevalence in the general population [1, 2]. SHC refers to complaints without a pathophysiological explanation or where the pathological findings are disproportionate to the illness experience [3]. The complaints can be very troublesome, affecting the ability to function both at work and in social settings.

Non-specific musculoskeletal complaints and mental health complaints present a major public health problem and a high economical burden in western societies [4–6], and are the most frequent reasons reported for sick leave [7–9]. Sick leave is a multi-causal phenomenon and there are different opinions regarding which factors are most important for sick leave (e.g. [10–13]). However, there is considerably more consensus regarding the negative consequences of long-term sick leave, both in terms of the major costs for society and organisations and the serious consequences it may have for the individual (e.g. [5, 14, 15]). Accordingly, it is important to gain knowledge about effective interventions to prevent and reduce long-term sick leave - both from a societal and an individual perspective.

Preventing the occurrence of SHC is difficult, or may not even be possible. These common complaints seem to be inherent in human nature and a part of everyday life, regardless of society or modern civilisation [16–18]. However, it may be possible to influence the employees’ perception and management of SHC, which in turn can have impact on sick leave and return to work after sick leave [19].

### Non-specific musculoskeletal disorders

Non-specific musculoskeletal disorders refer to pain or discomfort where it is not possible to identify an underlying cause of the pain, and back pain (BP) is the most common musculoskeletal complaint [20]. A multitude of treatments have been developed for the prevention of BP, but the results have been disappointing [20]. It seems difficult to prevent acute non-specific BP, but the consequences of the BP, such as fear of injury or activity, inactivity, and/or sick leave may be prevented [20]. Development of maladaptive perceptions about the cause and prognosis of BP is associated with a poorer clinical outcome [21]. The prevention of the negative consequences of BP can thus be seen as a way to improve the long term work participation for employees with BP, as well as decreasing the risk of the BP becoming chronic.

Brief Interventions (BI), based on the ‘non-injury model’ proposed by Indahl [22–26], have been among the most successful approaches to increase return to work for employees with BP [24, 25, 27–29]. According to this model, the spine is a strong and robust structure. Pain is not a sign of injury to the spine caused by any wrongdoing or ‘inappropriate’ behaviour. When a patient has the perception that the BP is caused by an injury to the spine and that the spine is likely to deteriorate with activity, inactivity is a rational choice. In the BI this illness perception [30] is challenged by presenting a perception of BP as a painful, but benign and usually self-limiting condition. The treatment providers’ job is not to ‘cure’ the pain, nor to remove fear of activity, but simply to present the evidence for the benefit of being active [31] and let the employee decide how to make best use of the information. The intention is to replace any maladaptive previous perceptions of BP. This non-injury model is consistent with the understanding and recommendations in the European Guidelines for the prevention of BP [20].

### Common mental disorders

Anxiety and depression are often termed ‘common mental disorders’ (CMD), because of their high prevalence, affecting 20–25 % of the adult population [32–34]. CMD has emerged as a major public and occupational health problem in many countries [5, 35]. Depression and mild anxiety are the most common mental disorders among employees [35, 36]. As with other mental disorders, the core symptoms of anxiety and depression affect a person’s emotional, cognitive and social functioning, which also may have impact on the capacity for work [37]. The increase in sick leave and work disability because of CMD has serious negative health and economical consequences calling for prevention [38–40]. Although mental disorders has become one of the greatest new social and labour market challenges in the OECD countries, little is known about the underlying causes of this phenomenon [9]. The most straightforward explanation would be an increase in the prevalence of mental disorders, but that does not seem to be the case. Most of the studies that have examined this, find limited evidence to suggest an increase in the prevalence of mental disorders over time (e.g. [41–45]). It appears that the increased awareness of complaints that have always been there without really being acknowledged, also has led to more exclusion from the workforce for these problems [9].

There is a high degree of comorbidity between CMD and BP [46–48]. In the general population persons with BP are more likely to report CMD than persons without BP [48, 49], and few pathological findings by physical examination in patients with BP are associated with more psychiatric symptoms than for patients with an identified structural or organic cause for the BP [50, 51]. However, the relationship seems to work both ways; BP can precede CMD, and CMD can precede BP [52]. Interventions targeting both BP and CMD should consider the high comorbidity between these conditions.

There is evidence that cognitive behavioural therapy and psychoeducational treatment for risk groups and individuals in an early stage of anxiety and depression may be effective [53–56]. However, reaching the majority of the population who are at risk of these disorders are difficult, because most people do not seek help until their problems are well advanced or do not seek help at all [57, 58]. Thus, population-based health promotion and prevention interventions targeting CMD may be useful, because it may be provided to everyone at risk, including those with no or very low risk. Population-based interventions are also found to be the most cost-effective interventions [59]. The workplace is an ideal setting for such population-oriented prevention programs [60, 61]. Distributing knowledge about CMD at the workplace, expressed with respect for the participants, delivered in a way that normalises but at the same time does not trivialise the complaints, presents an opportunity to overcome widespread stigma and fears concerning CMD. Stigma and self-stigma is still prevalent across the OECD countries [9].

Social support and coping skills are important factors to increase resistance to development of mental disorders [54, 62, 63]. Prolonged stress activation as a result of lack of coping might lead to feelings of helplessness and hopelessness, both proposed as cognitive models of depression [64–66]. Coping seems to be a stronger predictor for health than socioeconomic status [67, 68], and interventions aimed at targeting these factors can be expected to produce benefits to employees’ mental health, and further induce a beneficial effect on organisational health. Interventions providing information about mental health and illness report significant gains in knowledge, improved health, greater confidence in seeking help and providing help to others, decreased stigmatising attitudes, increased use of positive coping strategies, and improved social skills [69–74].

### The atWork intervention

atWork was established in 2007 as a new stepped-care approach to musculoskeletal complaints [19]. The intervention consisted of three workplace information meetings about BP to all employees, in addition to peer support. The atWork intervention targeting BP reduced sick leave and myths about BP in a randomised controlled trial (RCT) [19]. After this RCT the atWork intervention has been further developed with the goal to increase effect on health related measures. Because of the high comorbidity between BP and CMD, the high prevalence and the negative consequences of CMD, the atWork intervention has been modified to also comprise mental health complaints. A management seminar is also included, aiming to increase manager involvement and knowledge about the message distributed in the intervention.

atWork is a cognitive workplace intervention, based on the BI and the non-injury model [23–25]. atWork uses the workplace as an arena for health promotion. By focusing on altering employees’ beliefs and behaviour through evidence-based health information, atWork aims to enable employees to cope with the consequences of their health complaints [19]. This is done by providing insight and understanding of BP and CMD to all employees and managers, based on the non-directive social support model [75] and peer support [19]. atWork also has a theoretical foundation from the Cognitive Activation Theory of Stress (CATS), where coping is defined as a positive response outcome expectancy, a belief that your actions or strategies will lead to a positive result [66]. In addition to reaching out to all employees with the intervention, the aim of atWork is to reinforce an organisational culture where workers with health complaints are accepted as part of the normal work environment.

## Methods/Design

### Aims and objectives

The main aim of this study is to investigate if modifying the atWork intervention to also comprise a management course and knowledge about mental health complaints will improve the effect on sick leave and other health related outcomes compared to the original atWork intervention. We aim to address the following questions:

1. Is the modified atWork intervention more effective than the original atWork intervention in terms of reducing sick leave?
2. Is the modified atWork intervention more effective than the original atWork intervention in terms of increasing coping expectancies, job satisfaction and social support?

### Participants and recruitment

Eligible participants are private kindergartens, working with children from 0–6 years, in four Norwegian counties. In these four counties outpatient clinics and the necessary collaboration for implementing atWork are already established, so for convenience reasons we selected these counties for the trial. The first atWork trial was conducted on workplaces in the public sector [19]. This trial will investigate the effect of atWork on workplaces in the private sector. In Norway women have a higher sick leave rate than men, and a higher prevalence of SHC [1, 7]. The kindergartens have a high percentage of women employed, and are therefore chosen as participants in this trial. Participants will mainly be recruited through The National Association of Private Kindergartens, but also through the Norwegian Labour and Welfare Administration (NAV) and Vestfold Hospital Trust.

### Interventions

**Fig. 1:**
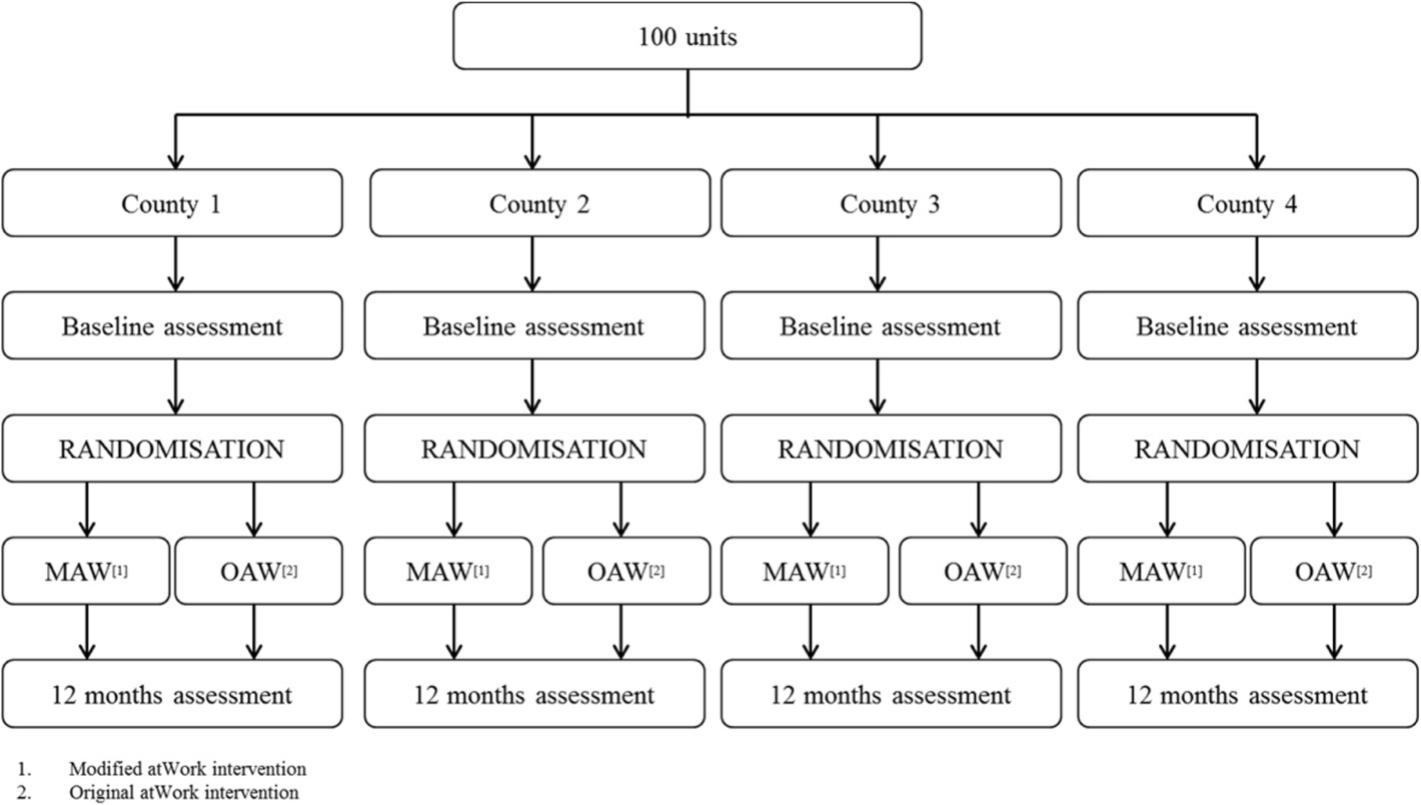
Flowchart of trial design

The participating kindergartens will be randomly allocated to one of two groups, receiving different workplace interventions aiming to increase participation in working life and prevent sick leave (see Fig. 1). One group will receive *the original atWork intervention (OAW)*, and the other group will receive *the modified atWork intervention (MAW)* [19]. We firmly believe that one of the success criteria of the atWork intervention is that all employees get the same information. We therefore recommend that managers encourage all employees to attend the workplace sessions and facilitate their attendance. To ensure a high participation rate, the different workplace sessions will be held several times at the same workplace if necessary. All workplace sessions will last for approximately one hour, and will be conducted by healthcare workers from Vestfold Hospital Trust.

1. The original atWork intervention, OAW
  The OAW intervention consists of three workplace sessions for all employees, and peer support [19].

#### Workplace sessions

The workplace sessions are for all employees at the workplace, also the managers. The first workplace session focuses on the prevalence of BP, what non-specific health complaints are, what the atWork intervention is and the reason for its development, and the selection of a peer adviser (see below). Questions are encouraged.

The second workplace session focuses on giving evidence-based information on spine and pain physiology, and how to cope with BP. The main message is the non injury model and the evidence for it, and emphasising the importance of staying active when experiencing BP. Questions and discussions on how the workplace may accommodate employees with BP are encouraged.

The third workplace session focuses on the quite widespread myths about BP, such as the consequence of inactivity and bed rest, or the value of imaging like X-rays and MRs. Questions are encouraged.

#### Peer support

Peer support involves selecting a ‘peer adviser’ at each workplace. The peer adviser is a fellow worker, recruited among employees in each kindergarten during the first workplace session. Recruitment takes place either by volunteering or agreeing after being suggested by colleagues. All peer advisers will participate in two seminars at an outpatient clinic. The seminars will focus on more in depth knowledge about the spine and BP, and guidance on how to function as a peer adviser at the workplace. The peer advisers’ role is to give social support and to use their local knowledge of the working environment to help their colleagues stay at work despite the BP. The peer advisers are instructed not to give any medical advice or to recommend treatment options. If an employee has persistent BP, or is unsure about the nature of the BP, the peer adviser will guide the employee to make an appointment with their general practitioner. All peer advisers will be given contact information to the outpatient clinic, and may at any time contact healthcare workers for general help or help with specific cases. The peer advisers will also receive a booklet with information, and a book explaining BP in layman’s terms [76].

All seminars in the OWA will be conducted by healthcare workers from Vestfold Hospital Trust.

2. The modified atWork intervention, MAW
  The MAW intervention includes one session for the managers and two workplace sessions for all employees. After these three sessions there will also be one additional session for the managers for the purpose of reflection and review of the content and use of the information presented in the intervention.

#### Session for managers

The first session is for managers’ at all organisational levels, health and safety representatives, and local union representatives, as these positions may function as facilitators for a good psychosocial workplace environment. The session provides an introduction to the atWork intervention, and informs the participants about what will be communicated to all employees. It is important that managers and workplace representatives understand and agree with the message distributed, to support the use of this knowledge at the workplace. The session also focuses on how to create a health promoting workplace perceived as welcoming to workers with health complaints, how to facilitate work for employees when needed, and where to get external support when needed. The support and consideration of managers is a strong determinant of job satisfaction and effective in alleviating employee strain in a wide variety of work settings [77]. These seminars will be conducted by healthcare workers from Vestfold Hospital Trust, together with a consultant from the resource center for an inclusive working life at NAV. The purpose of this collaboration is to increase the organisational knowledge about how to cope with health complaints in the work setting.

#### Workplace sessions

The two workplace sessions are for all employees at the workplace, including managers, health and safety representatives, and local union representatives. The first workplace session focuses on evidence based information about CMD, including prevalence, physiology, anxiety, stress, depression, comorbidity, myths, and coping. The information will emphasise that these complaints are experienced by many people, with the purpose of increasing inclusion and social support, and decreasing stigmatising attitudes. Questions and discussions on how the workplace may accommodate employees with CMD are encouraged.

The second workplace session focuses on evidence based information about BP, including prevalence, spine and pain physiology, myths, comorbidity, and coping. The main message is the non injury model and the evidence for it, and emphasising the importance of staying active when experiencing BP. Questions and discussions on how the workplace may accommodate employees with BP are encouraged. Number of sessions targeting BP is reduced in the MAW compared to OAW. This is due to a low attendance rate on the last workplace sessions in the first RCT [19], and participants’ feedback. Employees have expressed that three sessions targeting BP leads to a great deal of overlap and repetition, and experience this as a waste of time in a busy work schedule.

#### Reflection and review session

The reflection and review session is for managers’ at all organisational levels, health and safety representatives, and local union representatives, and will be a meeting where reflection on how to implement the new knowledge at the workplace is encouraged. The aim of this meeting is to discuss how each particular workplace can create an inclusive culture, and what further assistance they may need to achieve this goal. Further assistance will mainly be given by NAV, as a part of their daily work as a resource centre for inclusive working life.

Peer support is not a part of the MAW. In the OAW the peer adviser was not frequently used [19]. Companies have also reported that the role interferes with the management structure in the organisation, it takes too much time from work (the 2 days of qualification), and some of the tasks assigned to the role is perceived to collide with management responsibilities. The peer adviser is thus removed and a seminar for managers is added to the modified intervention.

### Inclusion and exclusion

All private kindergartens in the four counties Telemark, Vestfold, Buskerud, and Akershus are eligible for participation in the study. All employees in the kindergartens participating in the study are eligible for participating in the survey.

### Randomisation

The kindergartens will be randomised to one of the following two groups; 1) OAW or 2) MAW. This is done according to a computer generated randomisation list, generated by the trial statistician. The block randomisation is stratified by county and size of the kindergarten (small: <11 employees, large: ≥11). The randomisation and treatment allocation procedures are performed by a research technician at the randomising unit (Uni Research Health) and are concealed from the researchers and healthcare workers. The code for intervention allocation will not be revealed to the researchers or the healthcare workers until recruitment and baseline data collection are completed. The trial coordinator emails the randomisation unit with information about the name of the kindergarten, the county where it is located, and the size (small or large). Information about intervention allocation is emailed back. For obvious reasons there is no blinding to group assignment.

### Ethical considerations

The Regional Committee for Medical and Health Research Ethics for South-Eastern Norway has approved the study (Registration 2014/162/REC South East). The research will be carried out in compliance with the Helsinki declaration. The participants are informed about the study from their manager and from an information sheet at the start of the electronic survey. In the information sheet the participants are told that by continuing to the questionnaire after having read the information, they are giving their informed consent to participate in the study. In the information sheet the right to withdraw from the trial at any time without any explanation is emphasised. The timeframe for questionnaire completion is estimated to approximately 20 min. All participating kindergartens are thoroughly informed about the random allocation to either OAW or MAW. OAW has been effective in reducing sick leave. MAW contains crucial elements from the OAW, and is modified with the aim to increase positive effect on health related variables. The companies receiving OAW during the study period will be offered the sessions that are unique for the MAW after the project is terminated.

### Data collection

Survey data will be collected from both groups at baseline and 12 month follow-up. Data will be collected electronically using secure survey software (Qualtrics®). The baseline questionnaire is administrated by email to the manager at each kindergarten immediately after enrolment. This email contains detailed information on the study processes and purposes, and a link to the study survey. The manager distributes this information to all employees, who then may make an informed choice on whether to participate. Recruitment and collection of baseline data started in November 2014. Recruitment will continue until a sufficient number of kindergartens are enrolled. In the baseline questionnaire all employees will be asked to enter their email address. The email address will be used to link answers from the baseline questionnaire with the follow up questionnaire. Follow-up questionnaires will be administered electronically to participants who provide their e-mail address at baseline. Participant will be assigned code numbers, and all data will be treated confidentially. Printed questionnaires will be an option for participants who prefer this to filling in an electronic version of the questionnaire.

Sick leave will be collected at unit level, through register data from NAV. This allows for complete and objective data and will be collected every quarter, from all kindergartens, with no loss to follow-up. Data will be collected for the year before and after the intervention.

### Outcome measures

The primary outcome of this study is sick leave at unit level, collected through register data from NAV. One kindergarten equals one unit. Because sick leave is collected at unit level, we will collect data from all employees in the participating companies, not only the employees responding to the questionnaires.

The secondary outcomes, *coping expectancies*, *health*, *job satisfaction*, *social support*, and *workplace inclusion,* will be measured through validated questionnaires, in addition to demographics and belief about BP and CMD:

- *Coping expectancies* will be measured using the Theoretically Originated Measure of the Cognitive Activation Theory of Stress, TOMCATS [68]. TOMCATS is a newly developed scale, designed to measure response outcome expectancies as defined in the Cognitive Activation Theory of Stress (CATS) [66]. The scale consists of three factors, which represent the three response outcome expectancies in CATS: positive expectancy/coping, no expectancy/helplessness and negative expectancy/hopelessness.
- *Subjective health complaints* will be measured using the Subjective Health Complaints Inventory, SHC [78]. The SHC-inventory records complaints without asking for attributions or medical diagnosis. The selection of items is not based on any specific theory, but covers the most frequent health complaints and reasons for being seen by the general practitioner [3]. The inventory has five subscales; musculoskeletal pain, pseudoneurological complaints, gastrointestinal complaints, flu, and allergy complaints, and covers the period of the previous 30 days.
- *Psychological distress* will be measured by the Hopkins Symptom Checklist, HSCL-10 [79–81]. The HSCL-10 consists of 10 items derived from the widely used HSCL-25, a questionnaire designed to measure psychological distress, or, more specifically, mainly symptoms of anxiety and depression [82]. The 10 items includes feeling panicky, anxious, dizzy, tense, sleepless, sad, worthless, hopeless, fault within self, and finding everything is a burden.
- *Job satisfaction* will be measured using the Global Job Satisfaction, GJS [83–85]. The scale consists of 6 items to measure an employee’s general affective reaction to his or her job without reference to any specific facets.
- *Psychological demands, decision latitude and social support* will be measured using the Demand-Control-Support-Questionnaire, DCSQ [86, 87]. DCSQ is based on the Demand–Control Model by Karasek and Theorell [88] and the support dimension is added to the model by Johnson and Hall [89]. The scale consists of three subscales; demands, latitude and support.
- *Social support* will be measured using the 16-item Social Support Inventory (SSI) [90–93]. SSI will in this study be used to measure the participants’ perceptions of received directive and nondirective support in a workplace setting. The scale consists of four factors, with four items in each category; nondirective instrumental, nondirective emotional, directive instrumental and directive emotional.
- *Workplace inclusion* will be measured using the Workplace Inclusion Questionnaire (Sveinsdottir V, Fyhn T, Opsahl J, Tveito TH, Indahl A, Reme SE; Development of the Workplace Inclusion Questionnaire, in preparation). The questionnaire examines attitudes towards including employees with various health complaints and/or limitations at the workplace. The questionnaire consists of short case stories describing people with various common diagnoses, such as BP and CMD, as well as common social groups that may be discriminated for other reasons. Employees and managers are asked to indicate how well the various individuals fit into their workplace. Each case story has four questions. The first two items addresses how well the person in the case story would fit into their workplace. If the respondent does not think the person in the case story fits well or very well into their workplace, the third item addresses the main barriers for this reason. The fourth item asks about the respondents’ previous experience with colleagues or employees that are similar to the case story in question.

### Sample size estimation and power calculation

Our estimate of the sample size is based on the results from the project of Odeen et al. [19], where the same method of data collection was used. We will collect sick leave data at unit level from the participating kindergartens. Data will be analysed at the unit level, according to the principle of assessing effect of interventions at the same level that they are conducted [94]. The data collected will be clustered by companies. Registered number of sick-leave days may follow a Poisson distribution. A straightforward sample size calculation based on an assumption of normal based data is therefore not valid for our project. Thus, we based our sample size estimation on the results from Odeen et al. [19], since the units we will include in this study are comparable to the units in the study of Odeen et al. They showed a significant change of 11 % in sick leave with 42 and 48 units. We plan to include a minimum of 50 units in each intervention group. If we base our calculation on an assumption that change in sick leave follows a normal distribution, a decrease of 20 % in the MAW group versus the OAW group (from 9.0 to 7.2 %, SD = 3) and a significance level set to 0.05, will have a power of 0.84. 100 kindergartens are estimated to comprise approximately 1100 employees.

### Statistical analyses

#### Primary analyses of effect

A Generalised Linear Mixed-effects Poisson Model will be used to investigate a possible difference between the two intervention groups on sick leave. The model will estimate rate ratios and will account for the random variation in sick leave days between the participating units, measured repeatedly over time [19].

#### Secondary analyses of effect

Analysis of the secondary outcomes will be conducted on the individual level and will be based on changes from baseline. T-tests and chi-square tests will be used to investigate if there are significant differences between the two intervention groups.

## Discussion

The effect evaluation of the modified atWork intervention (MAW) is a large and comprehensive project. We have chosen a randomised controlled design to assess the effect of the MAW compared to the OAW, a design considered to provide the most reliable evidence on the effectiveness of interventions, because the used procedures reduce the risk of confounding factors influencing the results. When finished, the project it will provide evidence based information on prevention of long term sick leave, which may be of considerable benefit both from a societal, organisational, and individual perspective. The project will also generate knowledge on coping expectancies and social support, both strong predictors for health. However, if the MAW proves to be effective, the described study provides limited data to investigate why an effect occurred. Investigating more closely what the participants perceive as useful parts of the two interventions e.g. by focus group discussions, might add valuable insights to our research and intervention development.

The MAW is designed to further improve the effect on sick leave and other health related variables compared to the OAW. Mental health complaints is a frequent reason reported for sick leave and disability pension in Norway, and other interventions providing information about mental health and illness have reported positive outcomes such as decreased stigmatising attitudes, improved health and social skills, greater confidence in seeking help and providing help to others, and increased use of positive coping strategies. The OAW was effective in reducing sick leave and myths about BP, and our hypothesis is that adding information about mental health complaints to the intervention may produce additional positive effects.

## Abbreviations

BI: Brief intervention
BP: Back Pain
CMD: Common mental disorders
MAW: Modified atWork intervention
NAV: Norwegian Labour and Welfare Administration
OAW: Original atWork intervention
RCT: Randomised controlled trial
SHC: Subjective health complaints
TMG: Trial management group
TSC: Trial steering committee
